# Parotid Oncocytoma as a Manifestation of Birt-Hogg-Dubé Syndrome

**DOI:** 10.1155/2018/6265175

**Published:** 2018-06-03

**Authors:** Kazuki Yoshida, Masao Miyagawa, Teruhito Kido, Kana Ide, Yoshifumi Sano, Yoshifumi Sugawara, Hiroyuki Takahata, Nobuya Monden, Mitsuko Furuya, Teruhito Mochizuki

**Affiliations:** ^1^Department of Radiology, Ehime University Graduate School of Medicine, Toon, Japan; ^2^Department of Pulmonary Surgery, Ehime University Graduate School of Medicine, Toon, Japan; ^3^Department of Diagnostic Radiology, Shikoku Cancer Center, Matsuyama, Japan; ^4^Department of Pathology, Shikoku Cancer Center, Matsuyama, Japan; ^5^Department of Head and Neck Surgery, Shikoku Cancer Center, Matsuyama, Japan; ^6^Department of Molecular Pathology, Yokohama City University Graduate School of Medicine, Yokohama, Japan

## Abstract

Birt-Hogg-Dubé syndrome (BHD) is a rare autosomal dominant disease characterized by skin fibrofolliculomas, pulmonary cysts, spontaneous pneumothoraces, and renal cancers. Oncocytomas are benign epithelial tumors that are also rare. Recently, there have been a few case reports of BHD with a parotid oncocytoma that appears to have a BHD phenotype. Here we document the eighth known case and describe the magnetic resonance imaging features of the parotid oncocytoma, which mimicked Warthin's tumor. Radiologists should be aware of the association between these rare disorders.

## 1. Introduction

Birt-Hogg-Dubé syndrome (BHD) is a rare autosomal dominant disease characterized by skin fibrofolliculomas, pulmonary cysts, spontaneous pneumothoraces, and renal cancers [1]. In 1977, Birt, Hogg, and Dubé reported on a group of patients from single kindred who had multiple fibrofolliculomas with trichodiscomas and acrochordons [2]. This hereditary condition was later named Birt-Hogg-Dubé syndrome. In 2002, Nickerson et al. identified the BHD gene, which codes a protein called folliculin [3]. The BHD gene is now known as the folliculin gene (*FLCN*).

Patients with BHD often have renal tumors. Pavlovich et al. reported that 34 (27%) of 124 patients with BHD had renal tumors with variable histology, most commonly hybrid oncocytic tumors, and chromophobe renal cell carcinomas [4]. However, there are few reported cases of BHD with parotid gland oncocytoma. Here we present a rare case of a patient with this association.

## 2. Case History

**Figure 1:**
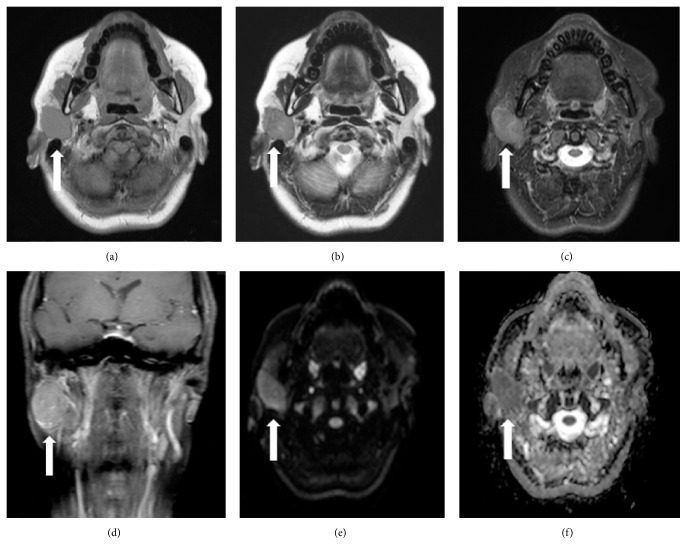
Magnetic resonance images of parotid oncocytoma. (a) T1-weighted imaging. (b) T2-weighted imaging. An axial magnetic resonance image shows a well-demarcated mass (white arrow) in the superficial and deep lobe of the right parotid gland. (c, d) The mass appears hyperintense to the native parotid gland tissue on fat-saturated T2-weighted imaging but hypointense on contrast-enhanced, fat-saturated T1-weighted imaging. (e, f) On diffusion-weighted images, the mass appeared hyperintense and hypointense according to the apparent diffusion coefficient.

A 44-year-old woman presented to our hospital complaining of right lower facial swelling and pain around the parotid gland. Her past medical history was unremarkable. However, she had a family history of pneumothoraces in her father and brother. Magnetic resonance (MR) imaging showed a 35 mm diameter mass in the superficial lobule of the right parotid gland. The lesion appeared hypointense on both T1-weighted MR imaging (T1WI) and T2-weighted imaging (T2WI). On fat-saturated T2WI, the masses appeared hyperintense when compared with the native parotid gland tissue but hypointense on contrast-enhanced T1WI with fat saturation. The lesion was hyperintense on axial diffusion-weighted imaging with a b-value of 1000 s/mm^2^ and a low apparent diffusion coefficient (Figure 1). The lesion was suspected to be Warthin's tumor, and a right superficial parotidectomy was performed accordingly.

**Figure 2:**
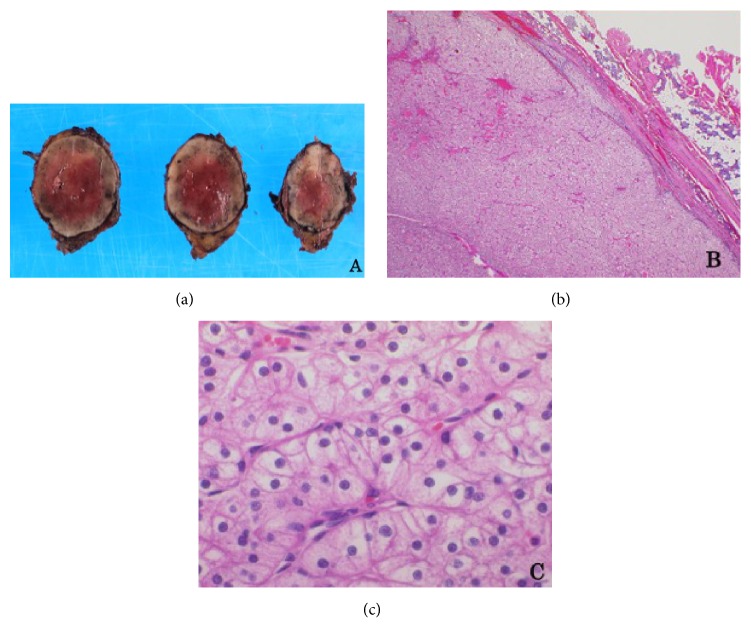
Histopathologic findings of parotid clear cell oncocytoma. (a) A well-circumscribed, solid, mahogany-colored nodule. (b, c) Microscopically, fibrous encapsulated nodules containing oncocytic tumor cells with a clear cytoplasm. (hematoxylin-eosin staining; original magnification, 20× in (b) and 400× in (c))

There was a well-circumscribed, solid, mahogany-colored nodule measuring 3.7 × 2.8 × 2.4 cm in the superficial parotidectomy specimen (Figure 2(a)). Microscopically, the nodule was an encapsulated tumor containing oncocytic cells. These cells formed solid clusters or trabecular patterns, separated by thin strands of fibrovascular stroma (Figure 2(b)) and were round in shape with centrally placed nuclei and clear cytoplasm. Neither necrosis nor capsular invasion was observed. Cytoplasmic granules enriched with glycogen were present but there was no mucin on periodic acid-Schiff staining (data not shown). Although the morphology was comparable to oncocytoma of the kidney, radiologic examination excluded the possibility of a metastatic renal tumor, and immunostaining for PAX-2 and CD10, which are markers for renal cell carcinoma, was negative (data not shown). The pathologic diagnosis was clear cell oncocytoma.

**Figure 3:**
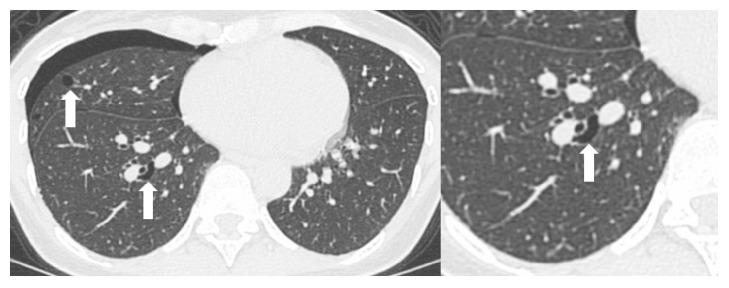
Pulmonary computed tomography demonstrating a right pneumothorax and multiple cysts (arrow). An ellipsoidal cyst abuts on the proximal portion of the pulmonary artery.

One and a half years later, the patient presented to hospital again, this time with mild dyspnea. Physical examination revealed decreased breath sounds on the right side. A computed tomography (CT) scan showed a right pneumothorax and multiple cysts. The cysts were located in the medial basilar regions of the lung fields bilaterally and were ellipsoid in shape and variable in size. Some of the cysts abutted the proximal portions of the lower pulmonary arteries (Figure 3). The patient was strongly suspected to have BHD and was subsequently referred for genetic counseling. Informed consent was obtained from the patient for* FLCN* genetic testing, which was performed on genomic DNA extracted from peripheral leukocytes. Duplication of cytosine was identified in the C8 tract of exon 11 (c.1285dupC), confirming the diagnosis of BHD. The patient's brother, who had an episode of pneumothorax, asked for genetic testing and was found to have the same mutation as the proband (data not shown).

## 3. Discussion

BHD is a rare disease and there are some reports of its prevalence. In North America, 102 BHD-affected families have been reported by the National Cancer Institute group [6, 15]. In Asia, 312 affected individuals from 120 Japanese BHD families have been reported [16]. However, information regarding the manifestations of BHD apart from pulmonary cysts, renal tumors, and cutaneous manifestations is limited. Oncocytomas are rare benign epithelial tumors, accounting for only 0.5%–1.5% of all salivary gland tumors. The parotid gland is the site most often affected, accounting for 78%–84% of salivary gland oncocytomas [12]. These tumors are slightly more prevalent in women than in men and usually occur in the seventh to ninth decades of life [14].

Lieu et al. reported the first case of BHD with parotid oncocytoma in a 56-year-old man [5]. Lindor et al. then reported a 45-year-old Caucasian woman with BHD who presented with multiple oncocytic parotid tumors [8]. Pradella et al. also reported a parotid oncocytoma that had arisen in a patient with BHD [9]. In a report by Schmidt et al. in 2005 on 219 BHD-affected individuals, four parotid gland tumors were documented in 2 men and 2 women. Three of those tumors were classified as oncocytoma [6]. In 2011, Maffé et al. reported on 19 BHD-affected individuals, including a 53-year-old man with bilateral parotid oncocytoma. There was a relative reduction of the wild-type signal from the parotid oncocytoma in this patient, who was heterozygous for the* FLCN* mutation. They reported that parotid oncocytoma should be considered as a component manifestation of BHD [7]. To the best of our knowledge, our patient is the eighth reported case of BND with parotid oncocytoma. The previous seven reports and our present case are summarized in Table 1.

*FLCN* encodes the protein folliculin (FLCN), which acts as a tumor suppressor. FLCN forms a complex with FLCN-interacting protein 1 (FNIP1) and FNIP1 homologue FNIP2, which interacts with 5'-AMP-activated protein kinase and regulates signaling of the mammalian target of rapamycin [17, 18].

The clinical characteristics of BHD are thought to be age-related. Skin lesions usually develop after the age of 20 years. Pulmonary cysts and pneumothoraces are often found in young adult patients aged 20–30 years, and renal cell tumors are more likely to develop after the age of 40 years. Therefore, BHD should be considered in patients who develop repeated spontaneous pneumothoraces and have skin fibrofolliculomas [1, 17]. Abdominal imaging is recommended at least every 3 years in the clinical follow-up of BHD. We prefer MR imaging to CT as it avoids exposure to radiation [18].

The MR imaging reports on parotid oncocytomas suggest that these lesions are hypointense on both T1WI and T2WI [10, 14], are hyperintense on diffusion-weighted imaging, and have low apparent diffusion coefficient values. Dynamic contrast-enhanced MR images show early enhancement with early washout [10]. Parotid oncocytomas also show high uptake of ^18^F-fluorodeoxyglucose on positron emission tomography (Table 2) [11]. Oncocytomas and Warthin's tumors have very similar features on imaging and therefore are very difficult to differentiate [19]. However, oncocytomas have unique imaging features that have led to them being known as “vanishing tumors” and may help in the diagnosis. Oncocytomas are hard to detect on fat-saturated T2 and T1 postcontrast MR images because of the similarity of intensity of the tumor and the parenchyma, hence the term vanishing tumor. Oncocytes have a low free water content and a lipid-rich membrane. If the amount of lipids in the tumor is approximately equal to that in the normal parotid gland tissue, the vanishing phenomenon occurs [13]. However, in our patient, the lesion appeared slightly hyperintense on fat-saturated T2WI and hypointense on contrast-enhanced and fat-saturated T1WI. The histology was that of a clear cell oncocytoma, which contains large amounts of glycogen and lipid that is different from that of normal oncocytes. A mixed oncocytoma/pleomorphic adenoma is hyperintense on fat-saturated T2WI and contrast-enhanced, fat-saturated T1WI [13]. These different MR imaging features may reflect the histopathologic findings. The previous seven case reports did not describe the imaging findings in detail. It is uncertain if the MR findings of solitary oncocytoma are different from those of oncocytoma associated with BHD; therefore, further investigation is needed.

Tobino et al. reported that multiple, irregular-shaped cysts of various sizes with lower medial lung zone predominance were characteristic features of BHD on CT [20]. Cysts abutting or including the proximal portion of the lower pulmonary arteries or veins are also highly probable in BHD (Figure 3). Our patient had these features, which were very helpful in making a diagnosis of BHD.

In conclusion, we describe the eighth confirmed case in the literature of parotid oncocytoma in BHD, which mimicked Warthin's tumor on MR imaging. Parotid oncocytoma appears to be one of the phenotypes in BHD. It is difficult to distinguish between oncocytomas and Warthin's tumors; however, radiologists should be aware of this association and consider parotid oncocytoma as a differential diagnosis if they detect a parotid mass similar to Warthin's tumor in BHD. If additional imaging is to be recommended, a dedicated renal-protocol MRI would be the choice, alone or in addition to chest CT.

## Figures and Tables

**Table 1:** Reported cases of parotid oncocytoma in Birt-Hogg-Dubé syndrome.

Case	Year	First author and region	Age at diagnosis, years /Sex	Mutation	Pathology of parotid tumor (Age at diagnosis)	Skin	Lung (Age, years)	Kidney (Age, years)	Family history
1	2000	Liu et al. [5] North America	56/M	N/A	Oncocytoma (55)	FF	PTX, LC	-	Sister (PTX, LC)

2-4	2005	Schmidt et al [6] North America	N/A	N/A	Oncocytoma	N/A	N/A	N/A	N/A

5	2011	Maffé et al. [7] Europe	53/M	c.347 dup A (exon 5)	Oncocytoma (32, right; 43, left)	FF or TD	PTX (42)	CCC (37)	Father (hybrid CCC/on with CCC component accounting for 60% of the lesion and sigmoid adenocarcinoma)

6	2012	Lindor et al. [8] North America	45/F	c.779+1 G>T (exon 7i)	Oncocytic neoplasm (45)	FF	-	-	Maternal grandfather (prostate cancer), maternal grandmother (bladder cancer, lung cancer)

7	2013	Pradella et al. [9] Europe	N/A	c.347 dupA (exon 5)	Oncocytoma	FF	PTX LC	N/A	N/A

8	2016	Present report Yoshida et al. Asia	45/F	c.1285dupC (exon 11)	Clear cell oncocytoma (44)	-	PTX (45) LC	-	Father (PTX) Brother (PTX)

M: male: F: female; FF: fibrofolliculoma; TD: trichodiscomas; PTX: pneumothorax; LC: lung cysts; CCC: clear cell carcinoma; N/A: not available.

**Table 2:** Imaging features of parotid oncocytoma.

No.	First author, year	Age/Sex BHD or not	Pathology	Size (mm)	CT enhancement	MRI T1WI	MRI T2WI	MRI DWI/ADC	MRI T2WI FS	MRI T1WI CE FS	FDG- PET
1	Kasai et al. [10] 2007	56/F	Onc	25	N/A	Hypo	Hypo	High /Low	Iso	Iso	N/A

2	Shah et al. [11] 2007	76/M	Onc	20	Homogeneous	N/A	N/A	N/A	N/A	N/A	Intense FDG uptake

3- 12	Tan et al. [12] 2010	49–74/ 7 female 3 male	Onc	6–66	6 Heterogeneous 4 Homogeneous	N/A	N/A	N/A	N/A	N/A	N/A

13- 21	Patel et al. [13] 2011	N/A 6 female 3 male	8 Onc 1*∗*	13–34	N/A	9 Hypo	N/A	N/A	8 Iso 1 Hyper*∗*	8 Iso 1 Hyper*∗*	N/A

22	Lindor et al. [8] 2012	45/F ***BHD***	Onc	9	N/A	Hypo	***Hyper***	N/A	N/A	N/A	N/A

23	Sepúlveda et al. [14] 2014	67/F	Onc	73	Heterogeneous	Iso	N/A	N/A	Hyper	Hyper	N/A

24	This report Yoshida et al.	44/F ***BHD***	Clear cell Onc	35	N/A	Hypo	Hypo	High /Low	***Hyper***	***Hypo***	N/A

M: male; F: female; Hypo: hypointense; Iso: isointense; Hyper: hyperintense; N/A: not available; T1WI: T1-weighted imaging; T2WI: T2-weighted imaging; DWI: diffusion-weighted images; ADC: apparent diffusion coefficient; T2WI FS: T2-weighted imaging with fat saturation; T1WI CE FS: postcontrast T1-weighted imaging with fat saturation; FDG-PET: ^18^F-fluorodeoxyglucose positron emission tomography; Onc: oncocytes. *∗*Mixed oncocytoma/pleomorphic adenoma.
